# Genome wide profiling of human embryonic stem cells (hESCs), their derivatives and embryonal carcinoma cells to develop base profiles of U.S. Federal government approved hESC lines

**DOI:** 10.1186/1471-213X-6-20

**Published:** 2006-05-03

**Authors:** Ying Liu, Soojung Shin, Xianmin Zeng, Ming Zhan, Rodolfo Gonzalez, Franz-Josef Mueller, Catherine M Schwartz, Haipeng Xue, Huai Li, Shawn C Baker, Eugene Chudin, David L Barker, Timothy K McDaniel, Steffen Oeser, Jeanne F Loring, Mark P Mattson, Mahendra S Rao

**Affiliations:** 1 Laboratory of Neurosciences, National Institute on Aging Intramural Research Program, National Institutes of Health, Baltimore, Maryland 21224, USA; 2 Buck Institute for Age Research, Novato, California 94945, USA; 3 Bioinformatics Unit, Branch of Research Resources, National Institute on Aging Intramural Research Program, National Institutes of Health, Baltimore, Maryland 21224, USA; 4 Burnham Institute for Medical Research, La Jolla, California 92037, USA; 5 Laboratory of Molecular Neurobiology, Medical Biochemistry and Biophysics, Retzius Laboratory, Karolinska Institute, Stockholm 17177, Sweden; 6 Illumina, Inc. San Diego, California 92121, USA; 7 Department of Neuroscience, School of Medicine, Johns Hopkins University, Baltimore, Maryland 21205, USA; 8 Corporate Research Laboratories, Invitrogen Corporation, 1620 Faraday Avenue, Carlsbad, CA 92008, USA; 9 Zentrum für Integrative Psychiatrie, Kiel Niemannsweg 147, 24105 Kiel, Germany

## Abstract

**Background:**

In order to compare the gene expression profiles of human embryonic stem cell (hESC) lines and their differentiated progeny and to monitor feeder contaminations, we have examined gene expression in seven hESC lines and human fibroblast feeder cells using Illumina^® ^bead arrays that contain probes for 24,131 transcript probes.

**Results:**

A total of 48 different samples (including duplicates) grown in multiple laboratories under different conditions were analyzed and pairwise comparisons were performed in all groups. Hierarchical clustering showed that blinded duplicates were correctly identified as the closest related samples. hESC lines clustered together irrespective of the laboratory in which they were maintained. hESCs could be readily distinguished from embryoid bodies (EB) differentiated from them and the karyotypically abnormal hESC line BG01V. The embryonal carcinoma (EC) line NTera2 is a useful model for evaluating characteristics of hESCs. Expression of subsets of individual genes was validated by comparing with published databases, MPSS (Massively Parallel Signature Sequencing) libraries, and parallel analysis by microarray and RT-PCR.

**Conclusion:**

we show that Illumina's bead array platform is a reliable, reproducible and robust method for developing base global profiles of cells and identifying similarities and differences in large number of samples.

## Background

Embryonic stem cells (ESCs), derived from the inner cell mass of pre-implantation embryos, have been recognized as the most pluripotent stem cell population. Human ES cells (hESCs) can be maintained and propagated on mouse or human fibroblast feeders for extended periods in media containing basic fibroblast growth factor (bFGF) [1-4] while retaining the ability to differentiate into ectoderm, endoderm and mesoderm as well as trophoectoderm and germ cells. Gene expression in hESC has been investigated by a variety of techniques including massively parallel signature sequencing (MPSS), serial analysis of gene expression (SAGE), expressed sequence tag (EST) scan, large scale microarrays, focused cDNA microarrays, and immunocytochemistry [5-7]. Markers for hESCs that may also contribute to the "stemness" phenotype have been established and markers that distinguish ESCs from embryoid bodies (EB) have been developed. Novel stage-specific genes that distinguish between hESCs and EBs have been identified and allelic differences between ESC have begun to be recognized [8-10].

As the potential of hESCs and their derivatives for regenerative medicine is being evaluated, it has become clear that the overall state of the cells, degree of contamination and comparisons of the more than a hundred different newly derived lines will need to be performed. It will be necessary to develop methods to monitor and assess hESC and their derivatives on a routine basis. Since differentiated cells are often scattered within or at the edge of colonies [11] and the differentiation is so subtle that morphological characteristics and even immunohistochemistry are insufficient to detect it, larger scale methods of analysis need to be developed.

Our strategy was to compare a variety of different hESC lines that were derived and expanded by three different institutions (WiCell Research Institute, BresaGen, Inc., and Technion-Israel Institute of Technology), and cultured in two separate laboratories (Burnham Institute and NIA) to a baseline set of data against which cell samples can be compared. By using cells grown in different conditions we expected to be able to identify core commonalities and by comparing feeders and embryoid bodies (EB) with hESC identify measures of contamination and early markers of differentiation. Further, by comparing embryonal carcinoma cell (EC) and karyotypically variant lines with hESC, we would be able to directly assess their utility as surrogates (for quality control purposes) for hESC.

We employed a pre-commercial prototype of the Illumina HumanRef-8 BeadChip [12], a genome-scale bead based array technology that combines the sensitivity and low cost of a focused array with the coverage of a large scale array, while requiring much smaller sample sizes than MPSS, EST scan or SAGE. We show that the Illumina bead based array correctly identified blinded duplicates as the closest related samples and readily distinguished between hESC lines, as well as between ESCs and EBs derived from them. This array allowed us to estimate the degree of feeder contamination present in the cultures. Similarities and differences between EC line NTera2 and hESC lines could be determined and verified, and the database comparisons allowed us to identify core self-renewal pathways that regulate hESC propagation.

## Results

### Multiple hESC lines can be assessed by Illumina bead array

Forty-eight samples were selected from multiple laboratories and gene expression profiles were examined using a bead array containing 24,131 transcripts derived from the Human RefSeq database that included full length and splice variants. Each gene was represented by sequences containing an average of thirty beads to provide an internal measure of reliability. Samples included 7 hESC lines BG01, BG02, BG03, I6, H1, H7 and H9, EBs that were differentiated from hESCs of the three BG lines, human fibroblast feeder HS27 (ATCC), hESC-derived fibroblasts, karyotypically abnormal hESC line BG01 Variant (BG01V) [13] and EC line NTera2 [14]. Samples were blinded and biological and technical repeats were examined at the same time. A single slide contained eight replicates and six such slides were used for the present set of samples. Results were normalized to average following Illumina Beadstudio manual and the quality of each sample was assessed by immunocytochemitsry and RT-PCR prior to subjecting them for analysis (data not shown). Results from the entire sample set are available for download as an excel spreadsheet (Additional file 1) and a CD of the results is available upon request. The total number of genes identified as expressed at >0.99 confidence is summarized in Table 1. Intensity results are reported in arbitrary units and ranged from 10 to 20,000 (a two thousand fold range). Although the sensitivity of the array has been reported to be high, in the present report we have restricted our analysis to expression of at least 100 units in any one sample. Using this cutoff, on average cells expressed approximately 8,000 transcripts (Table 1, 2), a number similar to the number detected by SAGE, MPSS and EST analysis [5-7,10,15,16]. As with other analysis, genes with the highest abundance were housekeeping genes, ribosomal genes and structural genes (Table 2 and Additional file 1). These genes were similar in most samples though relative levels varied.

**Table 1:** Correlation coefficients of paired samples in this bead array In order to test the reproducibility and reliability of the bead array, duplicate samples of hESC lines H9, I6, and EC line NTera2 and human fibroblast feeders (HS27) were run at the same time and correlation coefficients (R^2^) of duplicates were generated using the entire data of all genes with expression level >0 (§), or genes with detection confidence >0.99 (*), or genes with detection confidence >0.99 and expression level > 100 arbitrary units (#). Note that the correlation coefficients are in the range of 0.9382–0.9761 and the number of genes was in the range of 10,000–14,000.

Duplicate Samples	No. of all genes (expr.>0)^§^	R^2^of all genes (expr. >0)	No. of genes (>0.99)*	R^2 ^of genes (>0.99)*	**No. (>0.99, level>100)#**
H9	18,899	0.8681	13,672	0.9708	**7,408**
I6	19,139	0.8663	12,570	0.9761	**6,826**
NTera2	19,162	0.8741	14,036	0.9382	**7,147**
Feeder	18,157	0.8724	10,606	0.9751	**7,021**

**Table 2:** Distribution of genes with expression levels <50 and >50–10,000 as detected by Illumina bead array in 8 hESC populations All human ESC samples were hybridized in one experiment and the relative detection levels of genes were binned to obtain a global overview of transcription, approximately 8, 000 genes (~50%) were greater than 100 arbitrary units. The numbers are similar to results obtained by other large scale analysis such as MPSS.

Abundance (relative detection levels)	H9		H9 on human feeders		I6		BG01		BG02		BG03		BG01V		Pooled (H1, H7, H9)	
	
	No.	%	No.	%	No.	%	No.	%	No.	%	No.	%	No.	%	No.	%
<50	2,909	21.2	6,067	38.4	3,559	25.3	4,528	30.7	4,256	30.5	4,706	32.2	419	4.3	5,803	34.4
>50	10,817	78.8	9,747	61.6	10,484	74.7	10,232	69.3	9,694	69.5	9,915	67.8	9,438	95.7	11,085	65.6
**>100**	**8,126**	**59.2**	**7,539**	**47.7**	**7,409**	**52.8**	**7,703**	**52.2**	**7,496**	**53.7**	**7,430**	**50.8**	**7,230**	**73.3**	**8,217**	**48.7**
>500	3,077	22.4	2,947	18.6	2,626	18.7	3,065	20.8	2,950	21.1	2,852	19.5	2,851	28.9	2,941	17.4
>1000	1,629	11.9	1,625	10.3	1,490	10.6	1,638	11.1	1,589	11.4	1,517	10.4	1,566	15.9	1,554	9.2
>5000	248	1.8	257	1.6	256	1.8	256	1.7	263	1.9	262	1.8	275	2.8	251	1.5
>10000	90	0.7	94	0.6	112	0.8	92	0.6	100	0.7	107	0.7	101	1.0	94	0.6
Total No. of genes detected at >0.99 confidence	13,726		15,814		14,043		14,760		13,950		14,621		9,857		16,888	

**Figure 1:**
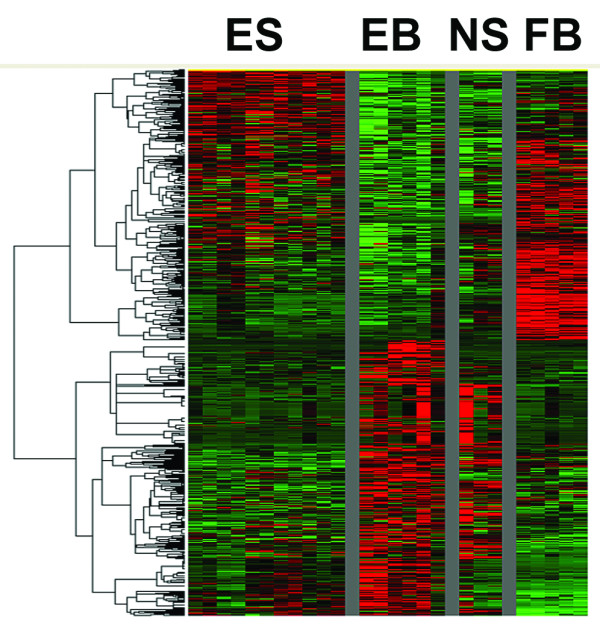
Unsupervised two-way hierarchical cluster analysis of differentially expressed genes illustrated in a heat-map. Each row represents the relative levels of expression of a single gene. Each column represents a sample. The samples include four groups of cells, ES designates 11 samples of hESCs, EB contains 6 samples of differentiated ESCs and EBs, NS consists of 3 hESC derived neural cells and FB is a collection of hESC derived mesenchyme and fibroblasts. High expressions relative to mean are colored red. Low expressions are colored green. Black represents no significant change in expression level between mean and sample. Samples cluster closer within their own group than samples from other groups.

**Figure 2:**
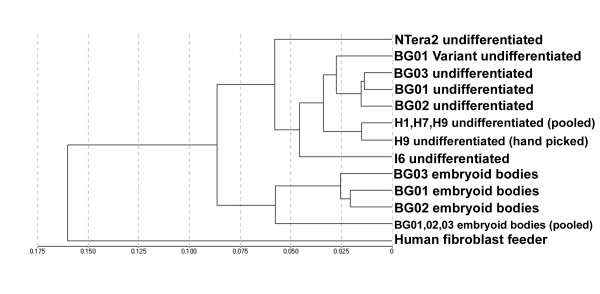
Dendrogram of unsupervised one-way hierarchical clustering analysis of relative expression of genes in selected samples. The clustering analysis was based on the average linkage and Euclidean distances as the similarity metric using differentially expressed genes identified by ANOVA (*p *< 0.05). hESCs clustered together and BG lines cultured in the same laboratory shared the largest similarities. EBs were separated from hESCs from which they were derived. EC line NTera2 and feeder cells can be distinguished from hESCs respectively.

One of the advantages of the Illumina arrays is the ability of running multiple samples simultaneously thus allowing multiple pairwise comparisons to be performed readily. To show the similarity of relative gene expression between samples, we have used Illumina Beadstudio and clustering software packages Pcluster [17] and TreeView [18] to generate a heat-map (Figure 1) and a dendrogram (Figure 2). Based on their properties, we classified some of our samples into four groups, (A) undifferentiated hESCs (including a sample from karyotypically abnormal variant, designated as "ES", n = 11); (B) differentiated ES cells and EBs (designated as "EB", n = 6); (C) hESC derived neural cells (designated as "NS", n = 3); and (D) hESC derived mesenchyme and human fibroblast feeder cells (designated as "FB", n = 5) and these groups were shown in the heat-map. Comparing the overall pattern of expression, we made several important observations: 1) Duplicates clustered close to each other and were more related to each other than to any other sample; 2) ESCs appeared more similar to each other than to EBs; 3) NTera2 cells appeared more similar to ESCs while differentiated NTera2 and EBs can be readily distinguished from their parent populations (Figure 2); 4) BG01V appeared similar to undifferentiated BG01 cells; 5) In general ESC lines grown in one laboratory appeared more similar than samples grown in other laboratories, suggesting that culture conditions affected gene expression but that this effect was much smaller than the effect of differentiation.

The global analysis suggested that the bead arrays used were sufficiently sensitive such that individual subsets of genes could be analyzed, different populations of cells could be readily distinguished and that a subset of candidate genes could be sufficient to distinguish between groups of cells. The comparison across multiple samples will allow a set of core stem cell markers to be identified. In subsequent sections we have performed such analysis. Readers are urged to analyze the expression of desired genes directly as it is impossible to test every gene given the large body of data generated.

### Comparison between MPSS and Illumina bead array results

We have previously used EST scan and MPSS to analyze pooled samples of ESCs and EBs from three different WiCell lines (H1, H7 and H9) [5]. Comparison between the two methodologies indicated that while there is good concordance for genes expressed at high levels, this does not hold for genes expressed at lower levels. As a test of the quality of the data generated in these experiments and to evaluate whether comparisons can be made across different methodologies, we re-ran the identical samples on the bead array platform. The complete comparison of gene expression is shown in Additional file 2 and is summarized in Tables 3 and Table 4. Overall, concordance in Illumina array was better than that evident between EST scan and MPSS datasets [9], but clearly showed much wider differences than that seen with running duplicates in the same assay format. Nevertheless, this comparison provides an independent verification of the data and suggests that if a sample is detected in more than one large-scale analysis, the reliability of the gene expression detection is high, which also reduces the number of individual genes needed to be verified. Caution should be observed in comparing different samples run on different platforms, especially when there has not been rigorous bioinformatic matching of the source sequences used to identify genes in the platforms. Often genes called by the same symbol originate from different database records, which may originate from different splice variants or contain sequence differences due to polymorphisms or outright error [19].

**Table 3:** Expression of hESC specific markers in pooled hESC sample as detected by Illumina bead array The expression of previously identified hESC markers was examined in all hESC samples (the values displayed represent the expression level of pooled H1, H7 and H9). Most of the genes were also identified using Illumina bead array in all 8 hESC populations in this study (1*), the gene CER1 was detected in all except one duplicate of H9 (2*), Nanog was not detected in all populations (3*) and Sox2, Lin41, NR6A1 and FoxD3 were not detected in the array although they were present in the chips for hybridization (4*).

Accession	Symbol	Pooled ES (H1, 7, 9)	Comments
NM_003641.1	IFITM1	8844	1*
NM_020997.2	LEFTB	6579.6	1*
NM_024674.3	LIN28	3944.1	1*
NM_175849.1	DNMT3B	3391	1*
NM_003212.1	TDGF1	3169	1*
NM_001769.2	CD9	2930.8	1*
NM_000165.2	GJA1	2404.4	1*
NM_021195.2	CLDN6	2247.2	1*
NM_004360.2	CDH1	1972.9	1*
NM_021127.1	PMAIP1	1601.5	1*
NM_032805.1	ZNF206	1504.5	1*
NM_003577.1	UTF1	1444.1	1*
XM_050625.2	SFRP2	1353.9	1*
NM_006548.3	IMP-2	1206	1*
NM_152312.2	GYLTL1B	1066.6	1*
NM_015973.2	GAL	1043.3	1*
NM_003240.2	EBAF	944.1	1*
NM_054023.2	SCGB3A2	890.5	1*
NM_020990.2	CKMT1	742.4	1*
NM_033668.1	ITGB1	694.7	1*
NM_003744.3	NUMB	618.3	1*
NM_007015.1	LECT1	597.4	1*
NM_021912.2	GABRB3	482.7	1*
NM_006729.2	DIAPH2	467.1	1*
NM_000222.1	KIT	188.9	1*
NM_005454.1	CER1	151.5	2*
NM_024865.1	NANOG	56.9	3*
NM_002701.1	POU5F1	694.4	1*
NM_003106	SOX2	ND	4*
NM_006458.2	LIN41	ND	4*
NM_001489.3	NR6A1	ND	4*
NM_012183	FOXD3	ND	4*

**Table 4:** Comparison of MPSS and Illumina bead array results The samples were analyzed by MPSS and bead array. The number of genes detected by each method and the degree of overlap is summarized. Note much higher degree of overlap when the top 2000 hits were compared. *: Most of the genes detected by MPSS were novel genes not included in the bead array.

ES	No.	%
Common in both (Top 2000 hits)	1,622	81.1
Common in both (All hits)	5,071	46.0
By bead array only	3,462	31.4
By MPSS only *	2,504	22.7

Total	11,037	100

EB	No.	%

Common in both (All hits)	5,168	43.1
By bead array only	4,131	34.4
By MPSS only *	2,694	22.5

Total	11,993	100

### Human feeders and hESCs can be readily distinguished and contamination can be readily assessed

**Figure 3:**
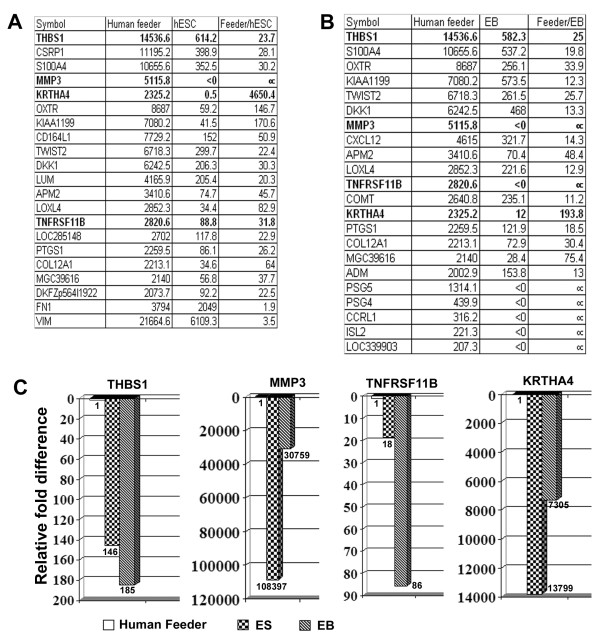
Human fibroblast feeder cells can be distinguished from hESCs and EBs. Bead array identified lists of genes that were uniquely expressed in human fibroblast feeders as compared to hESCs (A) and hEBs (B). The four genes whose expression was confirmed by qPCR (C) were in bold. In the graph (C), gene expression of each gene in feeder cells was designated as 1 fold and the bars represented fold decrease for each gene.

For all samples, we conducted an unsupervised one-way hierarchical clustering analysis. The clustering analysis was based on the average linkage and Euclidean distances as the similarity metric using differentially expressed genes identified by ANOVA (*P *< 0.05). The analysis revealed the underlying features and variation patterns of gene expression in each cell types. Figure 2 shows results of the cluster analysis of relative gene expression in selected samples. As one of our purposes of this study was to distinguish between human fibroblast feeders cells and hESCs and hEBs, wishing to readily detect feeder contamination in hESCs, we included one of the human feeder cells HS27 (ATCC) in this study. We have been using HS27 as feeder cells for H9 hESCs for more than two years and all hESCs grown on HS27 had normal karyotype, expressed all undifferentiated markers, and made teratomas with all germ layers (data not show). The global pairwise comparison clearly showed that human feeders were far more dissimilar to hESCs than hESCs grown in different laboratories, hESCs compared to their differentiated EBs that contained mesodermal tissue, and hESCs compared to the karyotypically variant hESC line BG01V. Pairwise comparisons of human feeders with hESCs resulted in a correlation coefficient of 0.66, which was less than the correlation coefficient of 0.71–0.74 observed between hESCs and their corresponding EBs. The large difference between human feeders and hESCs suggested that it would be possible to identify markers that were robust and reliable in distinguishing the two populations, and these markers would be sufficiently sensitive in detecting contamination of feeders. We examined the data to develop a list of genes that had high levels of expression in human feeder cells maintained in hESC medium but whose expression was low or absent in either ESCs or EBs. The absence of expression in EBs was used as a control for spontaneous differentiation of ESC colonies (including mesodermal differentiation) which may occur and the markers selected should be able to distinguish between these two events. A complete list of genes expressed at least ten-fold higher in human feeders is provided in Figure 3. Quantitative RT-PCR (qPCR) was used to verify the fold change of the expression of 4 genes, including THBS1, MMP3, TNFRSF11B and KRTHA4 (Figure 3C). Further confirmation can also be done using immunocytochemistry, as antibodies against these genes are commercially available.

Thus this comparison allowed us to distinguish between hESCs and human feeders and identify candidate markers that could detect feeder cell contamination should human feeders be used in the propagation of hESCs.

### hESCs and EBs can be distinguished from each other

Illumina bead array analysis confirmed that hESCs could be readily distinguished from EBs by global analysis. This raised the possibility that specific subsets of markers could be identified. We and others have used MPSS and EST scan and generated array data to make lists of hESC-specific genes [5,9,10,20]. As discussed above, most hESC markers identified by MPSS have been detected in the present bead array analysis (Table 3), confirming the utility of these previously identified markers for use in assessing undifferentiated status of hESCs. In addition, we have generated a list of genes differentially expressed at higher level in EBs than in hESCs, a subset of which is shown in Table 5. These markers were common to all EB samples tested and included genes known to be expressed in ectoderm, endoderm and mesoderm. The entire set of differentially expressed genes is provided in Additional file 3. Thus, the bead array format, which allows multiple pairwise comparisons, can be used to identify genes that are expressed by all differentiating EB samples in the present study. Our data suggested that a core set of limited markers might be sufficient to monitor the process of differentiation. By suitable selection of different germ cell layer specific markers one may also assess the overall quality of differentiation toward germ cells.

**Table 5:** Genes which are differentially expressed at higher levels in EBs than in hESCs

Symbol	All EB	All ES	EB/ES
RELN	1112	0.5	2224.0
SST	1394.2	5.5	253.5
SLC40A1	1210.1	9.1	133.0
IGF2	4896.9	38.4	127.5
SLN	2017.8	17	118.7
DCN	7588.1	76.2	99.6
ANXA8	4048.9	41.9	96.6
AQP1	1665.4	20.5	81.2
APOB	1256.4	15.8	79.5
AHSG	1414.3	20.8	68.0
NID2	1476.7	31.4	47.0
FGB	2210	52.4	42.2
LUM	8439.4	205.4	41.1
MGP	2772.4	69.8	39.7
THBD	1206.4	34.4	35.1
SERPINA1	1255.6	43.7	28.7
HAND1	12294	437.4	28.1
HBE1	1106.5	42.6	26.0
TTR	7661.2	347.1	22.1
HBG2	1601.1	85.6	18.7
COL2A1	1636.2	91.8	17.8
KIAA0977	1370.8	78	17.6
AFP	8941	552.3	16.2
COL3A1	13557	967	14.0
IGFBP3	8446.3	603.4	14.0
PAX6	1577.8	118.2	13.3
APOA1	9398.1	709.8	13.2
FRZB	3523.7	315.7	11.2
SPON2	1548.7	159.4	9.7
CEBPD	1100.2	122.7	9.0
DLK1	3355.8	374.8	9.0
RDC1	1589.7	192.1	8.3
BMP4	1851.5	227.3	8.1
PITX2	1057.9	131.2	8.1
ACTA2	5045	629.8	8.0
GAS1	1215.6	154	7.9
AGTRL1	1053.8	135.1	7.8
COL5A1	5876.4	765.4	7.7
CDKN1C	3134.1	412.9	7.6
CXCL14	2220.5	312.6	7.1
DOK4	1011.1	145.2	7.0
ARHGDIB	1635.3	246.5	6.6
FLRT2	1879.5	314.3	6.0
MSX1	2771.8	499.7	5.5

### Smaller but distinct differences among undifferentiated hESC lines

**Figure 4:**
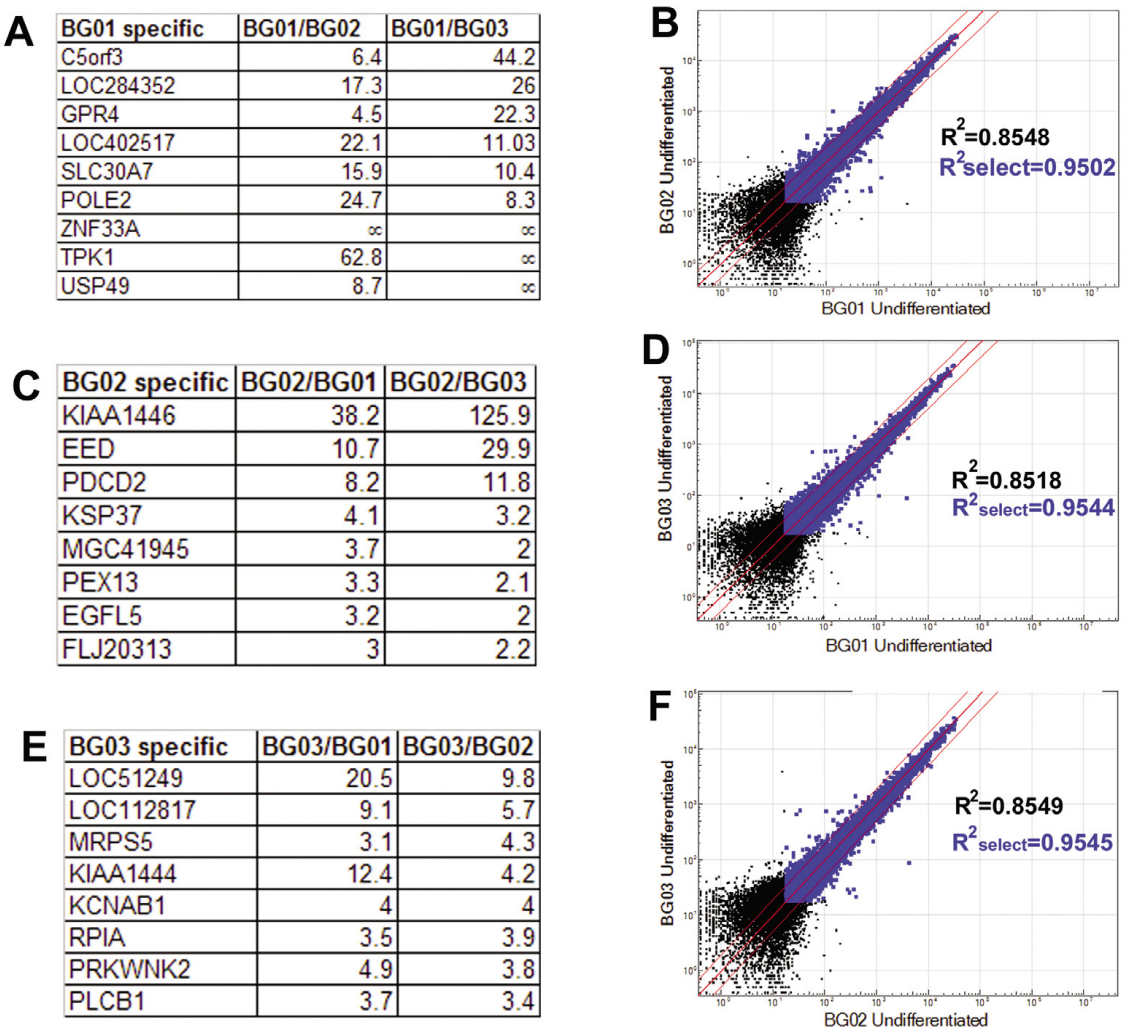
BG lines show small but distinct differences as assessed by bead array. These three hESC lines share high similarities as shown by the scatterplots of BG01 vs BG02 (B), BG01 vs BG03 (D) and BG02 vs BG03 (F). Comparisons of all three lines were made and lists of selected genes that were specifically expressed in BG01 (A), BG02 (C) and BG03 (F) are shown. Correlation coefficients (R^2^) were generated using all genes with expression level >0 (black and blue dots), or all genes with detection confidence >0.99 (blue dots). Genes outside the two thin red lines were detected at >2.5- fold difference.

Our cluster analysis indicated that BG01, BG02 and BG03 cell lines were overall more similar to each other than to other lines (Figure 1 and 2), but nevertheless showed additional differences than technical or biological repeats of the same sample. This raised the possibility that this microarray strategy may be sufficiently sensitive to identify relatively cell type specific candidate genes that could be used to distinguish one hESC population from another or to identify differences that were due to varied isolation and growth conditions. As a test we looked for differences between BG01, BG02 and BG03, which were grown in the same laboratory under the same conditions. Lists of candidate genes are shown in Figure 4A, C and 4E and the comparison of these three lines are shown in scatter plots in Figure 4B, D and 4F.

**Figure 5:**
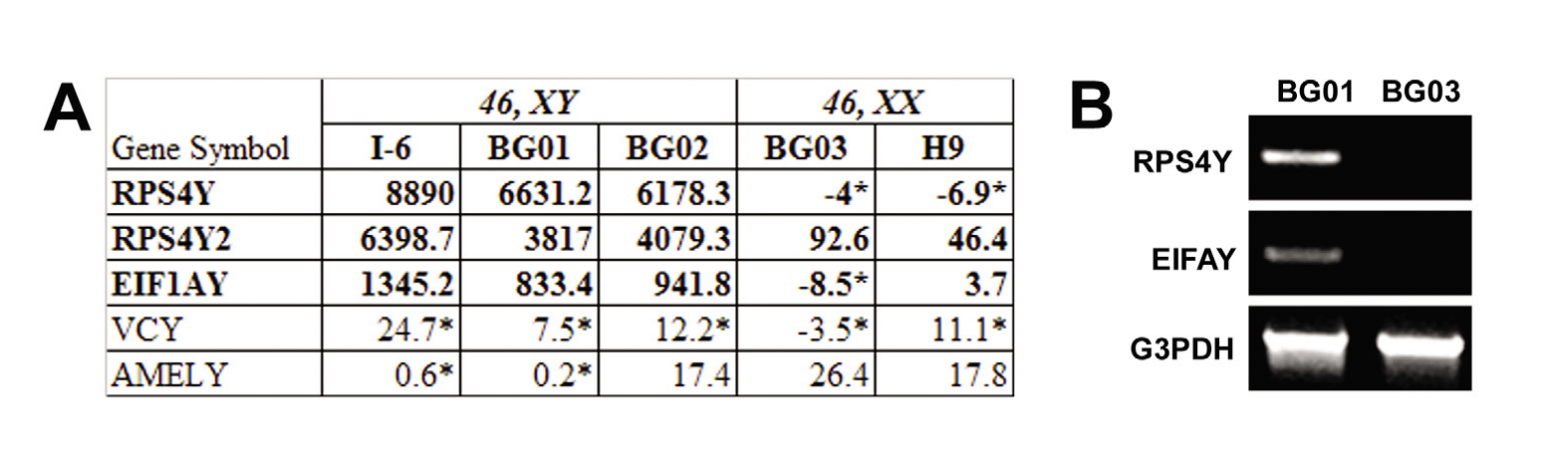
Male and female hESC lines can be distinguished by genes identified by bead array. Five potential genes RPS4Y, RPS4Y2, EIF1AY, VCY, and AMELY are located in the Y chromosome. By comparing the expression level of these genes in all hESC lines, we have found that 3 out of 5 were specifically expressed in male hESC lines I6, BG01 and BG02 (A) and this was verified by RT-PCR in male line BG01 and female line BG03 (B). G3PDH was used as an internal control. *: represents the gene expression level is detected at <0.99 confidence.

We reasoned as well that such a global comparison should allow us to distinguish between male and female lines if genes present on the Y chromosome were expressed at high levels in the undifferentiated state and were detected by the bead array. Several such candidate genes were identified. The most robust were RPS4Y, RPS4Y2, and EIF1AY (Figure 5). To confirm that these were useful markers, we designed RT-PCR primers and tested their expression in a male (BG01) and a female (BG03) line (Figure 5B). We noted that several of these continued to be expressed at high levels as ESCs differentiated to form EBs and upon further differentiation (data not shown), suggesting that these markers might be used in adult stem cell and germ cell populations as well.

In summary, our data suggest that the bead array format is sufficiently sensitive and global that it can distinguish one cell line from another even if those two cell lines are grown in the same laboratory under virtually identical conditions. Bead array can also be used to distinguish between male and female lines.

### Comparison of diploid pluripotent cells with NTera2 and BG01 variant

**Figure 6:**
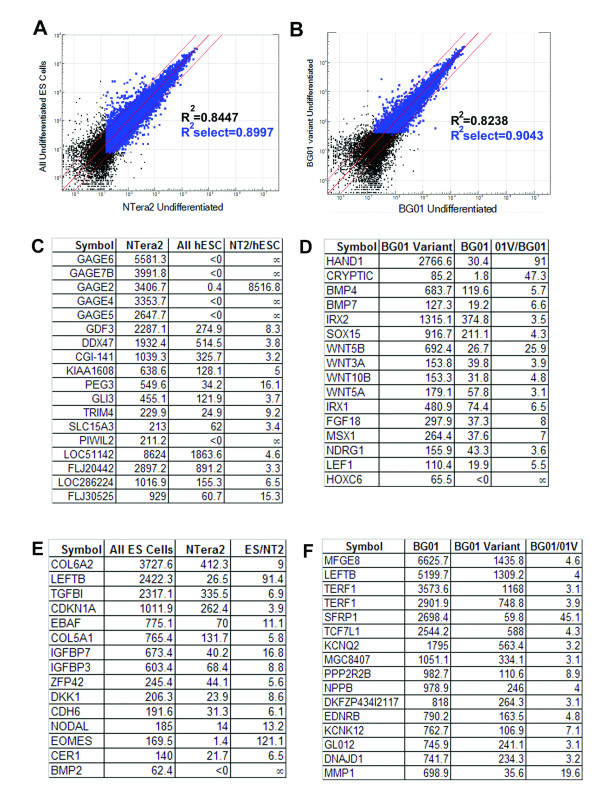
Diploid pluripotent EC cell line NTera2 and karyotypically abnormal hESC line BG01V can be distinguished from normal hESCs using Illumina array. Comparison of NTera2 and pooled hESC sample resulted a correlation coefficient of 0.8997. Two lists of genes, which were specifically expressed in NTera2 (C) or in hESCs (E) were identified. Likewise, while sharing similarities with BG01 (B, correlation coefficient= 0.9043), BG01V was different from BG01 in expression for many genes, particularly genes from the TGFβ pathway (D, F). Black dots represent genes that were detected at >0 expression level, blue dots represent genes that were detected both at > 0 expression level and at >0.99 confidence. Genes plotted outside the two thin red lines were detected at >2.5- fold difference.

Our previous results have suggested that EC lines share many of the properties of hESCs and can be used as a useful model for initial testing of biological questions [21]. More recently we have identified BG01V as a karyotypically abnormal variant that behaves much like its normal counterpart BG01, but is not subject to the same constraints of use as karyotypically normal hESCs [13]. Given the sensitivity of the bead array analysis, we tested its ability to detect the overall similarities and differences between NTera2 and a pooled ESC sample or between the karyotypically abnormal BG01V and its normal parent line (Figure 6).

Our results showed that, while NTera2 shared a high similarity with hESCs [21], it did have important differences with hESC lines. Examining these differences (summarized in Figure 6C and 6E), we noted that some reflected the origin of the tumor cells from which this line was derived [14]. Several germ cell markers such as GAGE2, GAGE7 and GAGE8 were highly expressed in NTera2 but were absent (or present at low levels) in any of the hESC lines examined (See Figure 6C and Additional file 1. Note that the GAGE genes are highly similar in sequence, making it difficult to distinguish one family member from another through hybridization; thus, while all of these GAGE gene probes gave positive signal, it is difficult to say if the signal came from the specific gene itself or from cross-hybridization from one of the other family members). None of these were present in BG01V, indicating that the karyotypically abnormal variant is not the equivalent of a teratocarcinoma line such as NTera2. In addition to the expression of germ cell markers, we noticed a significant difference in the expression of genes in the TGFβ pathway, such as GDF3 (Figure 6C), TGFBI, CDKN1A, IGFBP7, IGFBP3, NODAL, CER1 and BMP2 (Figure 6E). This is consistent with the postulated role of this pathway in germ cell differentiation [22,23] and suggests that TGFβ pathway cannot be reliably tested using NTera2 as a model for hESC.

The BG01V showed clear differences from its normal counterpart and some major changes are summarized in Figure 6D and 6F. Early markers of differentiation appeared to be present at higher levels in BG01V as compared to any of the hESC lines examined, although hESC specific genes continued to be expressed at high levels (see Additional file 4). In particular, the Wnt pathway and the TGFβ signaling pathway (Figure 6D), both of which involved in the early process of differentiation [24,25], appeared to be activated (Additional file 4), suggesting that the role of growth factors and signaling in these early events cannot be readily studied in this cell line.

In summary, the analysis highlighted the utility of the potential reference standards NTera2 and BG01V, demonstrated their general similarity and provided detail on potential caveats to their application.

### Global arrays provide a snapshot of the state of the cells and identify core self-renewal pathways

We have utilized a small fraction of the data to demonstrate the overall utility of this approach and its sensitivity in identifying small differences in cell populations. An additional potential application of such an analysis is the ability to examine the general state of a particular signaling pathway and determine whether it is active. By comparing across many samples, a procedure previously expensive and difficult in terms of the RNA and replicate requirement, one can rapidly identify key regulatory pathways.

**Figure 7:**
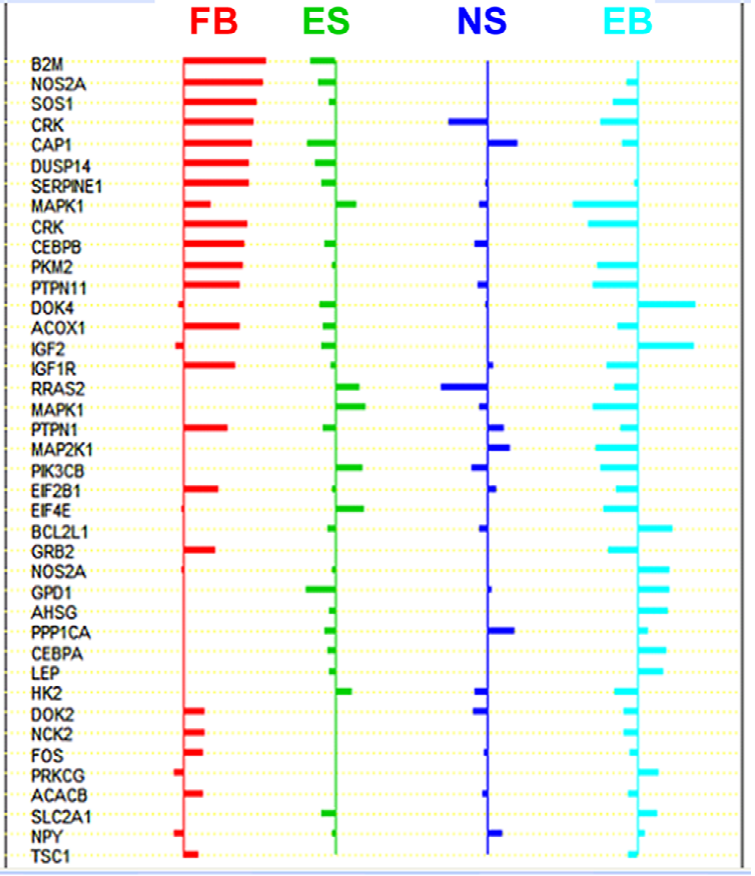
Identification of diagnostic markers by PAM. The shrunken class centroids for genes which have at least one nonzero difference are shown. The genes with nonzero components in each class were almost mutually exclusive and were the candidate molecular markers for the diagnosis of the four groups of cell populations, including, (from left to right) hESC derived mesenchyme and human fibroblast feeder cells ("FB", n = 5), undifferentiated hESCs ("ES", n = 11), hESC derived neural cells ("NS", n = 3), and differentiated ES cells and EB, ("EB", n = 6). The identified biomarkers can be used to distinguish the four groups of cell populations.

To test whether we could use such multiple pairwise comparisons to elucidate the major regulatory pathways that may be required for hESC self-renewal, we examined several metabolic pathways. The results of the analysis of the insulin/insulin-like growth factor (IGF) signaling pathway are shown in Figure 7. Using the same 4 groups of samples as in Figure 1, we conducted PAM (Prediction Analysis of Microarray) [26], in search for biomarkers used in diagnostic identification of these four groups, ES, EB, NS, and FB. In PAM, a list of significant IGF pathway genes whose expression characterizes each diagnostic class was obtained. The average gene expression level in each class was divided by the within-class standard deviation. The nearest centroid classification computed took the gene expression profile from a new sample and compared it to each of these class centroids. For cross-validation of prediction results, multiple classification processes were performed on two data sets randomly constructed each time from the entire gene expression dataset. The first dataset, consisting of 70% of the total data, was used as the training dataset, and the other dataset, containing the remaining 30% of data, was used for the data prediction and verification process. The final biomarkers were determined in such a way that the misclassification error rate was minimal. The resulting graph (Figure 7) showed the shrunken class centroids for genes that had at least one nonzero difference in each diagnostic class. The genes with nonzero components in each class were almost mutually exclusive and represented candidate biomarkers for the diagnosis of each class. All data analyses were performed using the bioconductor package [17].

## Discussion

Undifferentiated hESCs have been analyzed by EST scan, MPSS, SAGE and microarray [5,10,16]. The goal of these experiments including our own is to develop a low cost reliable method to assess multiple samples to generate a global database of markers and to provide a method of identifying core measures of similarities and differences across multiple laboratories. We and others have proposed three alternative methods of assessment: Quantitative RT-PCR [9,20], focused arrays [27] or a large scale array with bioinformatics tools being utilized to focus on appropriate subsets of genes [5,7,15,16,28]. Each of these methods has its advantages and disadvantages. The present results suggest that the global Illumina bead array retains the advantages of low cost per sample associated with focused arrays yet still has the strength of the global attributes of MPSS or EST scan while requiring much less RNA and turnaround time.

To test this array format we examined samples from a variety of laboratories in a blinded fashion to determine whether the array was sufficiently sensitive and rapid for routine assessment. Duplicates using 100 ng of RNA were run and results obtained forty-eight hours later. The resolution was sufficient that ESC samples could be distinguished from one another and a variant karyotypically abnormal subclone could be distinguished from the parent population (correlation coefficient = 0.9043).

Aliquots of the pooled ES and pooled EB samples, which we had prepared for MPSS, were included in this run to compare these two methods directly. The current analysis confirms that comparison across platforms is difficult and that only positive results can be treated with any reliability. The absence of expression cannot be readily interpreted. In particular, genes expressed at low levels (greater than 70% of all genes detected) should not be assessed in cross platform comparisons. The limited concordance at low levels raises a question as to how many genes are actually expressed by any one cell line and whether the cutoff of 3 tpm used for MPSS or 100 intensity units for bead arrays is a reasonable cutoff. We used 100 units for our analysis and we would suggest that readers exercise similar caution.

Nevertheless even at this higher cutoff the arrays were remarkably sensitive and allowed us to readily distinguish between samples including cells grown in the same laboratory. The basis of the sensitivity could be attributed to a limited set of genes and those genes could be identified for future use. For example BG01V, while much more similar to BG01 than to any other cell type, could still be distinguished from a biological replicate of BG01 by the expression of a particular subset of differentiation markers (Figure 6). EC cells such as NTera2 could be distinguished from hESCs by the expression of germ cell markers and the presence of a partially inactivated TGFβ (BMP) signaling pathway (Figure 6).

Distinguishing ESCs from EBs was relatively straightforward. We have confirmed the utility of previously identified markers for use in this platform as well as identified an additional set of markers that can serve as biomarkers to distinguish between the hESC and EB states. A subset of these markers have been used to develop a qPCR assay that shows such a high sensitivity that changes in cell behavior can be detected after as little as twenty-four hours and the development of EBs can be reliably staged [10,20].

During the identification of ES and EB specific markers, we have noticed that some known hESC markers, such as Nanog, was not detected in all populations of hESCs that were included in this analysis. Several ESC-specific gene, including Lin41, Sox2 and FoxD3, were not detected in the array either (Table 3). We believe that the problem with Lin 41, Sox2 and FoxD3 is a technical one as we were able to confirm expression using alternate methods. We are in progress of redesigning appropriate probes for these genes. In the case of the gene Nanog, there are several pseudo genes in the genome for Nanog and it has been a major technical challenge designing primers or probes that are specific and sensitive. We believe that a partial explanation for the variability in Nanog expression is due to the lack of sensitivity to this gene. However, immunocytochemistry while not strictly quantitative shows similar variability when used to assess Nanog expression in different cell lines [9,27,28].

This large comparison between samples allowed us to identify markers that distinguish human feeder cells from hESC. While we have listed 19 potential markers (Figure 3) and identified several hundred potential markers as shown in Additional file 5, we suggest that as few as 3–4 genes may be sufficient. Previously we found that as few as four were satisfactory to distinguish between hESCs and hEBs, which are two much more closely related samples [9]. In this study we have confirmed by qPCR the differential expression of four genes, THBS1, MMP3, TNFRSF11B and KRTH4, to separate human fibroblast feeders and hESCs (Figure 3). Several markers such as MMP3 and TNFRSF11B have commercially available antibodies (R&D systems) that may be used to further confirm contamination of feeder cells by immunocytochemistry. Efforts to identify other useful antibodies based on these results continue [29].

While we have focused on the immediate utility of the Illumina array platform, it is important to remember that this array provides a global snapshot of cell state and the data obtained can be readily compared in order to determine key signaling pathways. The ability to compare multiple samples in one run enhances data selectivity and reliability. To make such analysis more readily available, we utilized several software tools including the software package available through Illumina. The BeadStudio software provided with the BeadLab and BeadStudio genetic analysis systems for use with the bead array datasets provides a useful set of analytical and presentation tools that allow straightforward comparisons, which are sufficient for average users. For detailed analysis we recommend using more specific commercial tools or software packages developed by NCBI.

## Conclusion

In summary, the Illumina bead array has several key strengths including high throughput, low cost and high sensitivity. By using this array, we can readily detect contaminating feeders and spontaneous differentiation, differentiate male and female lines and distinguish between one undifferentiated population and another. Such a global analysis allows us to assess context dependent signaling and identify biomarkers of particular states of cells. Our future efforts will focus on data mining and developing better cross platform comparison tools and generating focused high throughput arrays for quality control in clinical and research settings.

## Methods

### hESC culture

The hESC lines H1, H7 and H9 (WiCell, Madison, WI) were cultured on feeder layers derived from mitotically inactivated HS27 human fibroblast cells (HS27, ATCC), or mouse embryonic fibroblsts or under feeder-free conditions on Matrigel (BD, Franklin Lakes, NJ) coated plates for at least 10 passages. Culture medium for all cultures was composed of DMEM/F12-Glutamax 1:1, 20% Knockout Serum Replacement, 2 mM nonessential amino acids, 100 μM beta-mercaptoethanol, 50 μg/ml Pen-Strep (all from Invitrogen, Carlsbad, CA), and 4 ng/ml human recombinant basic fibroblast growth factor (bFGF/FGF2; PeproTech Inc., Rocky Hill, NJ.) Feeder-free cultures were prepared for gene expression analysis by manually harvesting individual colonies with uniform typical undifferentiated ESC morphology.

BG01 (46, XY), BG02 (46, XY), BG03 (46, XX), I6 (46, XY) and BG01V (BG01 karyotypic variant: 49, XXY, +12, +17): Cells were maintained for 3 (BG01V), 7 (BG02), 8 (BG01), or 21 (BG03) passages under feeder-free condition on fibronectin-coated plates in medium that had been conditioned by mouse embryonic fibroblasts for 24 hours. Culture medium was DMEM/F12, 1:1 supplemented with 20% Knockout Serum Replacement, 2 mM non-essential amino acids, 2 mM L-glutamine, 50 μg/ml Pen-Strep, 100 μM beta-mercaptoethanol, and 4 ng/ml of bFGF.

Different hESC lines were grown in slightly different culture conditions as described above. H lines were grown on Matrigel coated dishes, while BG lines on fibronectin treated dishes. These coating substrata supported the growth of hESCs similarly, as evaluated by colony morphology, immunocytochemistry and proliferation rate (data not shown).

Embryoid bodies (EBs) were prepared from BG lines as described in [5]. Cells were aggregated and cultured on non-adherent substrata for fourteen days.

#### Other cells

NTera2 cells were purchased from ATCC and cultured in parallel with hESCssamples using protocols described previously [21]. HS27 embryonic human newborn foreskin cells (ATCC CRL-1634) were grown in DMEM with 10%FBS.

All samples included in this study can be found in Additional file 6.

#### Bead array gene expression analysis

RNA was isolated from cultured cells using the Qiagen RNEasy kit (Qiagen, Inc, Valencia, CA). Sample amplification was performed using 100 ng of total RNA as input material by the method of Van Gelder et al [30]. Amplified RNA synthesized from limited quantities of heterogenous cDNA [30] was performed using the Illumina RNA Amplification kit (Ambion, Inc., Austin, TX) following the Manufacturer instructions. Labeling was achieved by use of the incorporation of biotin-16-UTP (Perkin Elmer Life and Analytical Sciences, Boston, MA) present at a ratio of 1:1 with unlabeled UTP. Labeled, amplified material (700 ng per array) was hybridized to a pilot version of the Illumina HumanRef-8 BeadChip according to the Manufacturer's instructions (Illumina, Inc., San Diego, CA). Amersham fluorolink streptavidin-Cy3 (GE Healthcare Bio-Sciences, Little Chalfont, UK) following the BeadChip manual. Arrays were scanned with an Illumina Bead array Reader confocal scanner according to the Manufacturer's instructions. Array data processing and analysis was performed using Illumina BeadStudio software.

#### Identification of differentially expressed genes and clustering analysis

Differentially expressed genes between ES and EB were identified by ANOVA at p value 0.05 using bioconductor [17]. Unsupervised hierarchical clustering analysis and principal component analysis (PCA) were conducted using software Pcluster [31] and TreeView [18].

#### Identification of diagnostic markers

PAM (prediction analysis of microarray) was employed for the identification of diagnostic markers from insulin pathway genes by using the software package bioconductor [17]. PAM is a class prediction method for expression data mining. It can provide a list of significant genes whose expression characterizes each diagnostic class. The average gene expression level in multiple classes, such as ES, EB, NS, and FB, was divided by the within-class standard deviation for that gene. The nearest centroid classification computed by PAM takes the protein expression profile from a new sample, and compares it to each of these class centroids [26].

#### RT-PCR and quantitative real-time PCR analysis

Total RNA was isolated with TRIzol (Invitrogen. cDNA was synthesized using 2.5 μg total RNA in a 20-μl reaction with Superscript II (Invitrogen) and oligo (dT)12–18 (Promega; Madison, WI). One microliter RNase H (Invitrogen) was added to each tube and incubated for 20 minutes at 37°C before proceeding to the RT-PCR analysis. The PCR primers are: RPS4Y-forward: 5' AGATTCTCTTCCGTCGCAG 3', RPS4Y-reverse, 5' CTCCACCAATCACCATACAC 3'; EIFAY-forward, 5' CTGCTGCATCTTAGTTCAGTC 3'; EIFAY-reverse 5' CTTCCAATCGTCCATTTCCC 3'. Quantitative real time PCR gene specific primer pairs and probes were purchased from Applied Biosystems (Foster City, CA) for the following genes: MMP3 (Hs00233962_m1), TFRSF11B (Hs00171068_m1), THBS1 (Hs00170236_m1), KRTHA4 (Hs00606019_gH), and for internal control β-actin (ACTB, Hs99999903_m1).

## Authors' contributions

YL contributed significantly in array data analysis and validation and drafted the manuscript. SS provided hESC samples and validated array results. XZ provided a large number of the hESC samples for this report and participated in manuscript drafting. HL performed bioinformatics study under the supervision of MZ who also participated in all discussions of the manuscript. RG and FJM isolated total RNA from the human ES cell line WA09 (H9) cell line grown on feeder (human HS27 fibroblast) and feeder-free conditions for > 20 passages at the Burnham Institute for Medical Research. These RNA samples were analyzed by Illuminas Bead Array under the supervision of JFL. CMS provided NTera2 sample, participated in analysis of this EC line and helped draft the manuscript. HX performed validation of the array results. SCB, EC, DLB, TKM and SO participated in the array design and analysis. MPM monitored the whole project. MSR conceived of the study, designed the whole project, coordinated the collaboration, supervised YL, SS, HX and CMZ, and helped draft the manuscript.

## Supplementary Material

Additional File 1 Gene expression of all samples included in this study. Click here for file

Additional File 2 Comparison of gene expression data of MPSS and Illumina bead array. Click here for file

Additional File 3 Genes that are specifically expressed in EBs as compared to hESCs. Click here for file

Additional File 4 Genes that are expressed more than 3-fold higher in BG01V than in BG01. Click here for file

Additional File 5 Genes that are specifically expressed in human fibroblast feeders as compared to hESCs and EBs. Click here for file

Additional File 6 List of all samples included in this study. Microsoft excel spreadsheet. Click here for file
