# Supplementary material for: Genome wide profiling of human embryonic stem cells (hESCs), their derivatives and embryonal carcinoma cells to develop base profiles of U.S. Federal government approved hESC lines
Source: BMC Dev Biol. 2006 May 3;6:20. doi: 10.1186/1471-213X-6-20 (PMC1523200; doi:10.1186/1471-213X-6-20)
Supplement: Additional File 3 — Genes that are specifically expressed in EBs as compared to hESCs. [file 1471-213X-6-20-S3.doc]

Pooled EB>ES (ES<0)

| OSF-2 | NM_006475.1 | osteoblast specific factor 2 (fasciclin I-like) (OSF-2). |  |  |  |  |  |
| --- | --- | --- | --- | --- | --- | --- | --- |
| SYNCOILIN | NM_030786.1 | intermediate filament protein syncoilin (SYNCOILIN). |  |  |  |  |  |
| SST | NM_001048.1 | somatostatin (SST). |  |  |  |  |  |
| DOC1 | NM_014890.1 | downregulated in ovarian cancer 1 (DOC1), transcript variant 2. |  |  |  |  |  |
| AGT | NM_000029.1 | angiotensinogen (serine (or cysteine) proteinase inhibitor, clade A (alpha-1 antiproteinase, antitrypsin), member 8) (AGT). |  |  |  |  |  |
| COL15A1 | NM_001855.2 | collagen, type XV, alpha 1 (COL15A1). |  |  |  |  |  |
| GAGEC1 | NM_007003.2 | G antigen, family C, 1 (GAGEC1). |  |  |  |  |  |
| FOXF1 | NM_001451.1 | forkhead box F1 (FOXF1). |  |  |  |  |  |
| RAI3 | NM_003979.2 | retinoic acid induced 3 (RAI3). |  |  |  |  |  |
| TCF21 | NM_003206.2 | transcription factor 21 (TCF21), transcript variant 2. |  |  |  |  |  |
| LOC253017 | XM_171068.2 | hypothetical protein LOC253017 (LOC253017). |  |  |  |  |  |
| MICAL2 | NM_014632.1 | flavoprotein oxidoreductase MICAL2 (MICAL2). |  |  |  |  |  |
| CLEC2 | NM_016509.1 | C-type lectin-like receptor-2 (CLEC2). |  |  |  |  |  |
| TF | NM_001063.2 | transferrin (TF). |  |  |  |  |  |
| SFTPD | NM_003019.2 | surfactant, pulmonary-associated protein D (SFTPD). |  |  |  |  |  |
| BDKRB1 | NM_000710.2 | bradykinin receptor B1 (BDKRB1). |  |  |  |  |  |
| RARB | NM_016152.2 | retinoic acid receptor, beta (RARB), transcript variant 2. |  |  |  |  |  |
| GSTA1 | NM_145740.1 | glutathione S-transferase A1 (GSTA1). |  |  |  |  |  |
| LOC375620 | XM_351747.1 | similar to aldo-keto reductase family 1, member B10; aldose reductase-like 1; aldo-keto reductase  family 1, member B11 (aldose reductase-like); aldose reductase-like peptide;  aldose reductase-related protein; small intestine reductase ... (LO |  |  |  |  |  |
| FLJ21075 | NM_025031.1 | hypothetical protein FLJ21075 (FLJ21075). |  |  |  |  |  |
| LY96 | NM_015364.2 | lymphocyte antigen 96 (LY96). |  |  |  |  |  |
| SLC2A5 | NM_003039.1 | solute carrier family 2 (facilitated glucose/fructose transporter), member 5 (SLC2A5). |  |  |  |  |  |
| KIAA0843 | NM_014945.1 | KIAA0843 protein (KIAA0843). |  |  |  |  |  |
| CYP4B1 | NM_000779.2 | cytochrome P450, family 4, subfamily B, polypeptide 1 (CYP4B1). |  |  |  |  |  |
| RAC1 | NM_018890.1 | ras-related C3 botulinum toxin substrate 1 (rho family, small GTP binding protein Rac1) (RAC1), transcript variant Rac1b. |  |  |  |  |  |
| PPBP | NM_002704.1 | pro-platelet basic protein (chemokine (C-X-C motif) ligand 7) (PPBP). |  |  |  |  |  |
| KRTHA4 | NM_021013.3 | keratin, hair, acidic, 4 (KRTHA4). |  |  |  |  |  |
| IL6 | NM_000600.1 | interleukin 6 (interferon, beta 2) (IL6). |  |  |  |  |  |
| KIAA0318 | XM_044334.4 | RIM binding protein 2 (KIAA0318). |  |  |  |  |  |
| WFDC3 | NM_181530.1 | WAP four-disulfide core domain 3 (WFDC3), transcript variant 4. |  |  |  |  |  |
| LOC51159 | NM_016206.1 | colon carcinoma related protein (LOC51159). |  |  |  |  |  |
| LOC56901 | NM_020142.3 | NADH:ubiquinone oxidoreductase MLRQ subunit homolog (LOC56901). |  |  |  |  |  |
| RNF29 | NM_184087.1 | ring finger protein 29 (RNF29), transcript variant 4. |  |  |  |  |  |
| HCF-2 | NM_013320.1 | host cell factor 2 (HCF-2). |  |  |  |  |  |
| REN | NM_000537.2 | renin (REN). |  |  |  |  |  |
| NLGN4Y | NM_014893.1 | neuroligin 4, Y linked (NLGN4Y). |  |  |  |  |  |
| FLJ20513 | NM_017855.2 | hypothetical protein FLJ20513 (FLJ20513). |  |  |  |  |  |
| ULBP2 | NM_025217.2 | UL16 binding protein 2 (ULBP2). |  |  |  |  |  |
| KLF5 | NM_001730.2 | Kruppel-like factor 5 (intestinal) (KLF5). |  |  |  |  |  |
| C8orf4 | NM_020130.2 | chromosome 8 open reading frame 4 (C8orf4). |  |  |  |  |  |
| SIM2 | NM_005069.2 | single-minded homolog 2 (Drosophila) (SIM2), transcript variant SIM2. |  |  |  |  |  |
| ATP8B4 | XM_370863.1 | ATPase, Class I, type 8B, member 4 (ATP8B4). |  |  |  |  |  |
| ASGR2 | NM_080914.1 | asialoglycoprotein receptor 2 (ASGR2), transcript variant 3. |  |  |  |  |  |
| LOC375737 | XM_351831.1 | similar to Ribosome biogenesis protein BMS1 homolog (LOC375737). |  |  |  |  |  |
| ITGA1 | NM_181501.1 | integrin, alpha 1 (ITGA1). |  |  |  |  |  |
| DKFZp761O2018 | XM_044062.7 | hypothetical protein DKFZp761O2018 (DKFZp761O2018). |  |  |  |  |  |
| RAB40B | NM_006822.1 | RAB40B, member RAS oncogene family (RAB40B). |  |  |  |  |  |
| LOC96626 | NM_033514.1 | pinch-2 (LOC96626). |  |  |  |  |  |
| LOC196051 | XM_113641.3 | hypothetical protein LOC196051 (LOC196051). |  |  |  |  |  |
| FZD1 | NM_003505.1 | frizzled homolog 1 (Drosophila) (FZD1). |  |  |  |  |  |
| AMHR2 | NM_020547.1 | anti-Mullerian hormone receptor, type II (AMHR2). |  |  |  |  |  |
| ITGBL1 | NM_004791.1 | integrin, beta-like 1 (with EGF-like repeat domains) (ITGBL1). |  |  |  |  |  |
| S100A3 | NM_002960.1 | S100 calcium binding protein A3 (S100A3). |  |  |  |  |  |
| SURB7 | NM_004264.2 | SRB7 suppressor of RNA polymerase B homolog (yeast) (SURB7). |  |  |  |  |  |
| SACS | NM_014363.3 | spastic ataxia of Charlevoix-Saguenay (sacsin) (SACS). |  |  |  |  |  |
| FLJ40288 | NM_173682.1 | hypothetical protein FLJ40288 (FLJ40288). |  |  |  |  |  |
| SERPINB8 | NM_002640.2 | serine (or cysteine) proteinase inhibitor, clade B (ovalbumin), member 8 (SERPINB8). |  |  |  |  |  |
| LOC116236 | NM_198147.1 | hypothetical protein LOC116236 (LOC116236). |  |  |  |  |  |
| CML2 | NM_016347.1 | putative N-acetyltransferase Camello 2 (CML2). |  |  |  |  |  |
| LOC390651 | XM_372599.1 | similar to olfactory receptor MOR245-8 (LOC390651). |  |  |  |  |  |
| COP | NM_052889.1 | CARD only protein (COP). |  |  |  |  |  |
| AKR1D1 | NM_005989.1 | aldo-keto reductase family 1, member D1 (delta 4-3-ketosteroid-5-beta-reductase) (AKR1D1). |  |  |  |  |  |
| ADORA2B | NM_000676.2 | adenosine A2b receptor (ADORA2B). |  |  |  |  |  |
| FMN2 | XM_371352.2 | formin 2 (FMN2). |  |  |  |  |  |
| TCF8 | NM_030751.2 | transcription factor 8 (represses interleukin 2 expression) (TCF8). |  |  |  |  |  |
| LACTB | NM_171846.1 | lactamase, beta (LACTB), nuclear gene encoding mitochondrial protein, transcript variant 2. |  |  |  |  |  |
| MYLK | NM_053031.1 | myosin, light polypeptide kinase (MYLK), transcript variant 7. |  |  |  |  |  |
| LOC375468 | XM_351629.1 | hypothetical gene supported by AK096941 (LOC375468). |  |  |  |  |  |
| KRT6B | NM_005555.2 | keratin 6B (KRT6B). |  |  |  |  |  |
| LOC376453 | XM_352228.1 | similar to 27 kDa Golgi SNARE protein (Golgi SNAP receptor complex member 2) (Membrin) (LOC376453). |  |  |  |  |  |
| GLRB | NM_000824.2 | glycine receptor, beta (GLRB). |  |  |  |  |  |
| Siat7c | NM_152996.1 | alpha-N-acetylgalactosaminide alpha-2,6-sialyltransferase III (Siat7c). |  |  |  |  |  |
| KLF12 | NM_007249.3 | Kruppel-like factor 12 (KLF12), transcript variant 1. |  |  |  |  |  |
| DPYD | NM_000110.2 | dihydropyrimidine dehydrogenase (DPYD). |  |  |  |  |  |
| LOC256394 | XM_170754.3 | similar to serine (or cysteine) proteinase inhibitor, clade A (alpha-1 antiproteinase, antitrypsin), member 4; protease inhibitor 4 (kallistatin) (LOC256394). |  |  |  |  |  |
| LHX2 | NM_004789.3 | LIM homeobox 2 (LHX2). |  |  |  |  |  |
| IL13RA2 | NM_000640.2 | interleukin 13 receptor, alpha 2 (IL13RA2). |  |  |  |  |  |
| LOC390158 | XM_372396.1 | similar to ribosomal protein L5; 60S ribosomal protein L5 (LOC390158). |  |  |  |  |  |
| DUSP10 | NM_144729.1 | dual specificity phosphatase 10 (DUSP10), transcript variant 3. |  |  |  |  |  |
| FLJ37818 | NM_175916.2 | hypothetical protein FLJ37818 (FLJ37818). |  |  |  |  |  |
| FLJ23306 | NM_024530.1 | hypothetical protein FLJ23306 (FLJ23306). |  |  |  |  |  |
| CD34 | NM_001773.1 | CD34 antigen (CD34). |  |  |  |  |  |
| IL7R | NM_002185.2 | interleukin 7 receptor (IL7R). |  |  |  |  |  |
| HOXA9 | NM_002142.3 | homeo box A9 (HOXA9), transcript variant 2. |  |  |  |  |  |
